# Post-Sternotomy Complications: Aortic Erosion and Pseudoaneurysm Formation Caused by Fractured Sternal Steel Band Wire

**DOI:** 10.1016/j.jaccas.2025.103375

**Published:** 2025-04-23

**Authors:** Casey Zachariah, Kathrin Freystaetter, Wolfgang Dietl, Dominik Wiedemann

**Affiliations:** a Karl Landsteiner University of Health Sciences, Krems, Austria; b Division of Cardiac Surgery, University Hospital St Poelten, St Poelten, Austria

**Keywords:** aorta, complication, computed tomography

## Abstract

**Introduction:**

A 66-year-old man with a history of homograft implantation in aortic position 30 years ago, was referred with an actively bleeding sternotomy wound.

**Case Summary:**

Computed tomography revealed perforation of the ascending aorta by the most cranial steel band wire, forming a large pseudoaneurysm. Surgery in deep hypothermic circulatory arrest included wire removal and pseudoaneurysm repair. On the 1st postoperative day (POD), significant mediastinal bleeding required revision surgery, leading to ascending aorta replacement. The patient fully recovered and was discharged on the 33rd POD.

**Potential Pitfalls:**

Conventional resternotomy was deemed too risky, even with cardiopulmonary bypass (CPB) support. Instead, peripheral CPB cannulation, cooling to 18 °C and resternotomy with the patient under circulatory arrest were the method of choice. Left ventricular (LV) venting via a left-side minithoracotomy prevented LV distention, which was critical owing to mild-to-moderate aortic valve incompetence and risk of ventricular tachycardias.

**Take-Home Message:**

Repair of the pseudoaneurysm was achieved safely; however, these procedures should be performed in high-volume centers specialized in aortic surgery.

## Visual Summary

**Figure:**
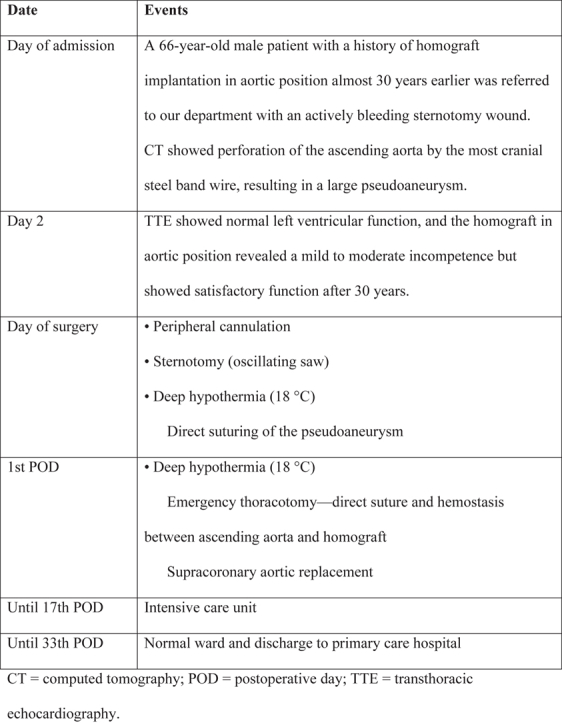

A 66-year-old male patient was referred to our department with a proximal sternotomy wound defect, which was oozing bloody fluid. He was referred to us after primary presentation in another cardiac surgical unit because he was operated on at our division 30 years before. In 1995 he received an aortic homograft due to aortic valve incompetence. Because a bleeding sternotomy wound almost 30 years after surgery is atypical and therefore alarming, the patient was admitted to our ward and a computed tomography (CT) scan was performed. This scan revealed a pseudoaneurysm of the ascending aorta with the most cranial sternal steel wire band penetrating the aneurysm sack, with a patent communication to the skin (Videos 1 and 2). This CT image depicts the perforation of the sternal band wire through the aorta and the thereby resulting pseudoaneurysm formation. Transthoracic echocardiography showed normal left ventricular (LV) function, and the homograft in aortic position revealed a mild to moderate incompetence but showed satisfactory function after 30 years. The aortic erosion and subsequent pseudoaneurysm formation was caused by a fractured steel wire band used for initial sternotomy closure. Because of the actively bleeding pseudoaneurysm of the ascending aorta, urgent surgical intervention was performed.

**Take-Home Messages**

• The combination of peripheral cannulation, DHCA, and LV venting via a minithoracotomy were the key to success.
• The course of the current patient shows that these procedures should be performed in high-volume centers with special experience and focus in aortic surgery.

## Case Summary

Peripheral cardiopulmonary bypass (CPB) was installed via the right subclavian artery and percutaneous cannulation of the right femoral vein. An LV vent was placed via an anterior minithoracotomy in the 5th intercostal space to ensure adequate left ventricular (LV) unloading. Deep hypothermic circulatory arrest (DHCA) was achieved at 18 °C before sternotomy.

After re-sternotomy and quick limited dissection of the relevant structures, the entry and exit points of the eroded steel wire band could be located. Both perforation sites were closed directly with pledgeted 2/0 Prolene sutures and showed no residual leaks. The patient was rewarmed to normothermia, the vent was removed, and the apex oversewn.

During the weaning process on the first postoperative day (POD), the initial course was uneventful, allowing for liberation from mechanical ventilation further leading to successful extubation. But immediately after extubation, 500 mL of blood spontaneously emptied from the mediastinal drain. The patient then became hemodynamically unstable, requiring increasing vasopressor doses. A large hematoma compressing the right atrium was seen on transesophageal echocardiography, and the decision for urgent revision surgery was made. A bleeding point was located between the proximal ascending aorta and the homograft. It was oversewn with 4/0 Prolene pledgeted sutures. Owing to fragility of the tissue the decision to replace the ascending aorta with a 30-mm Dacron prosthesis was made and the proximal aortic anastomosis was reinforced with Teflon felt. A routine postoperative CT scan on the following day showed no further abnormalities.

After weaning from sedatives, the patient initially showed no adequate responsiveness within 48 hours, so a cranial CT was conducted, revealing multiple small circumscribed cortical and subcortical primary ischemic areas on both frontal and occipital lobes. A tracheostomy was performed due to the suspected prolonged duration of ventilation, but the patient finally awoke initially presenting with discrete weakness in the lower extremity that completely resolved over time. The patient could then be successfully decannulated and transferred to the regular ward on the 17th POD. Further postoperative management mainly consisted of logopaedic and physical therapy. The patient could be discharged to his primary care hospital for further mobilization and care on the 33rd POD with no residual neurologic deficits.

The patient was readmitted to our ward 36 days after the initial discharge with suspected mediastinitis. However, further evaluations, including follow-up CT, did not confirm that diagnosis. Instead, elevated inflammatory markers in the blood were attributed to a reaction in the shoulder joint after an infiltration procedure performed in an outpatient setting. A routine follow-up was scheduled for six months; however, the patient opted not to attend this appointment. Instead, a telephone consultation was conducted to confirm his well-being.

## Procedural Steps

### Intervention 1

1. Peripheral CPB + DHCA
2. LV vent (anterior thoracotomy)
3. Median sternotomy (oscillating saw)
4. Direct closure of perforation sight (pledgeted 2/0 Prolene sutures)
5. Sternotomy closure (10 sternal wires)

### Intervention 2

1. Peripheral CPB + DHCA
2. Supracoronary ascending aortic replacement (30-mm Dacron)

## Potential Pitfalls

Conventional re-sternotomy was deemed far too risky even if performed with the patient on CPB support, so we performed peripheral CPB cannulation and cooled the patient to 18 °C; in this way re-sternotomy could be performed with the patient in circulatory arrest. In addition, LV venting was performed via a left-side minithoracotomy to avoid LV distention. This was especially important in this case due to the mild to moderate aortic valve incompetence and the increased risk of cardiac distention, ventricular tachycardias, and pulmonary edema.

Surgical techniques can vary greatly depending on location and etiology of the pseudoaneurysm. In this case, an immediate ascending aortic replacement could have been an alternate approach. However, the primary strategy was to aim for the least invasive technique to minimize the risk of redo surgery and to keep the period of circulatory arrest short, which is why initially the pseudoaneurysm was excluded with the use of direct sutures. In the preoperative CT, entry and exit sites of the steel band wires were well delineated and therefore presumed to be easily closed with the use of pledgeted sutures. Owing to the unforeseen postoperative complications, the patient finally required an ascending aortic replacement.

## Conclusions

Complications after median sternotomy are reported with a prevalence of approximately 0.5%-5%.^1^ The most common complications include mechanical complications (wire fracture, rotation, migration), osseous complications (fracture, dehiscence), and infectious components (osteomyelitis, abscesses, mediastinitis).^1^

These complications can range from insignificant to complex postoperative findings, such as presented in this case. Although it is extremely rare for such a complication to arise nearly 30 years after the initial surgical intervention, we assessed this case as urgent, due to the pseudoaneurysm's direct contact with the sternum.

Pseudoaneurysms are rather rare complications of aortic surgery, although some studies have documented an incidence of up to 7.7%.^2^ Mechanisms of these can include trauma, infection, technical issues relating to the anastomosis, and aortic tissue disease.^2^

To our knowledge this is the first case report illustrating such a complication 30 years after primary surgery. Repair of the pseudoaneurysm of our patient could be performed safely. However, several considerations regarding the management should be addressed in advance.

**Visual Summary:** Timeline of the Case

Date	Events
Day of admission	A 66-year-old male patient with a history of homograft implantation in aortic position almost 30 years earlier was referred to our department with an actively bleeding sternotomy wound. CT showed perforation of the ascending aorta by the most cranial steel band wire, resulting in a large pseudoaneurysm.
Day 2	TTE showed normal left ventricular function, and the homograft in aortic position revealed a mild to moderate incompetence but showed satisfactory function after 30 years.
Day of surgery	• Peripheral cannulation • Sternotomy (oscillating saw) • Deep hypothermia (18 °C) Direct suturing of the pseudoaneurysm
1st POD	• Deep hypothermia (18 °C) Emergency thoracotomy—direct suture and hemostasis between ascending aorta and homograft Supracoronary aortic replacement
Until 17th POD	Intensive care unit
Until 33th POD	Normal ward and discharge to primary care hospital

CT = computed tomography; POD = postoperative day; TTE = transthoracic echocardiography.

## Funding Support and Author Disclosures

Dr Wiedemann is a scientific advisor, consultant, or lecturer for Fresenius, Edwards, Abbott, and Octapharma. All other authors have no relationships relevant to the contents of this paper to disclose.

## Appendix

Video 1 Preoperative computed tomography, sagittal plane

Video 2 Preoperative computed tomography, axial plane
